# Person, Population, Mechanism. A Rejoinder to Critics and an Elaboration of the Three-Branch Model

**DOI:** 10.17505/jpor.2024.26295

**Published:** 2024-05-23

**Authors:** Lars-Gunnar Lundh

**Affiliations:** Department of Psychology, Lund University

**Keywords:** persons, populations, mechanisms, psychological science, person-sensitivity, generalizability, causal complexity, agency

## Abstract

In a previous paper (Lundh, 2023), it was argued that psychological science can be seen as having three main branches, corresponding to three levels of research: research at the person level, at the population level, and at the mechanism level. The purpose of the present paper is to discuss the critique that has been raised against this model by Lamiell (2024) and Nilsson (2024) and to elaborate and specify the three-branch model in more detail. This is done by an incorporation of Nilsson’s concept of person-sensitivity into the model, and by a clearer differentiation between the two contrasts involved: (1) the *methodological* focus either on individual persons or on populations of individuals; and (2) the *theoretical* focus either on whole-person functioning or on sub-personal mechanisms.

## 1. Introduction

In a previous paper (Lundh, 2023), it was argued that psychological science can be seen as having three main branches: person psychology, population psychology, and mechanism psychology. The basic suggestion was that psychological science involves research at three different levels: (1) the person level, (2) the population level, and (3) the sub-personal mechanism level. The person-level was described as focusing on psychological phenomena as experienced and enacted by *individual persons* in their interaction with other persons and other parts of the environment. The population level in contrast has its focus on *populations of individuals*, the frequency of various psychological phenomena in a population, and population-level effects of various psychological interventions. The mechanism level focuses on the explanation of psychological functioning in terms of *sub-personal mechanisms*. In the present paper this altogether is referred to as the *three-branch model* of psychological science.

Several researchers (Hofmann et al., 2024; Lamiell, 2024, Nilsson, 2024) have commented on my previous paper, and some of them have also raised criticisms of the three-branch model that deserve discussion. The purpose of the present paper is to respond to their critique and at the same time to elaborate further on the three-branch model. The next section of the paper (section 2) is devoted to a discussion of the critique raised by Lamiell (2024), who questions the very notion of “population psychology.” Although I agree with much of Lamiell’s reasoning, I will defend the claim that important psychological research is also done at the population level.

Another kind of critique is raised by Nilsson (2024), who argues that the three-branch model is not as clear-cut as it might seem. This is the topic of section 3. Nilsson also introduces the interesting concept of person-sensitivity and argues that it may be better to speak of *degrees* of person-sensitivity than of a separate *level* of person psychology. This calls for a more penetrating discussion of the entire three-branch model, which is the subject of sections 4 and 5.

One basic difficulty in these kinds of discussions is what Block (2000) speaks of as the “jingle and jangle” of psychological terms, where the same term is sometimes used for quite different psychological constructs (“the jingle fallacy”) and different terms are sometimes used for the same (or almost the same) psychological construct (“the jangle fallacy”). Although a certain terminological vagueness may be unavoidable (and perhaps even fruitful at a certain stage of the discussion when new ways of thinking are introduced), it is important to strive for terminological clarity, which may sometimes also require the introduction of new concepts.

## 2. In Defense of Population Psychology

According to Lamiell (2024), there is no such thing as a “population psychology.” As he argues, the knowledge from population-level studies, whether correlational, experimental, or mixed, is inherently *demographic* and not psychological in nature. Lamiell’s argumentation involves several claims, of which I agree with some and disagree with others. In the following three subsections, I will discuss three of his claims, of which I agree with the first one and disagree with the two others.

Section 2.1 focuses on Lamiell’s (2024, p. 61) critique of “the assumption of epistemic continuity” in the paradigm shift from individual-oriented research to population-level research that took place in the early 20th century. This is a critique which I fully share. In section 2.2, however, I will argue against Lamiell’s claim that causal processes only occur in individual beings and not in populations. And finally, in section 2.3, I will present some arguments for why some population-level research *does* belong to psychological science and *not* to demography.

In other words, my reasoning here represents a *defense* of population-level research as having a rightful place in psychological science, in combination with a *critique* of the assumption that population-level research is designed to provide knowledge about individual persons. There are two aspects of the critique against population-level research, and its role in present-day psychological research, that deserves to be repeated:

- There is a serious *problem-method mismatch* when researchers try to answer questions about *individual persons* by research on *populations*; and
- Population-level research has an inordinately large place in present-day psychology, whereas person-level research has a far *too limited* role.

### 2.1. The historical turn from person-level to population-level research

Psychological science underwent a radical change in the early 20th century, when the classic Wundt-ian experimental research paradigm with its focus on the individual was replaced by a statistical paradigm using questionnaires and focusing on populations of individuals. Despite this radical change, as Lamiell describes it,

the belief was broadly embraced at the time (and continues to prevail today) that the treatment group model offers an alternative means to the same overall knowledge objective as the original Wundt-ian model, i.e., the objective of advancing our scientific understanding of the psychological doings of individuals. (Lamiell, 2024, p. 62)

This is precisely the kind of thinking that was criticized in my original paper (Lundh, 2023) as an example of *the problem-method mismatch that results when researchers try to answer questions about individual persons by research on populations*. Population-level research, whether experimental or correlational, is not well suited to answer questions about individuals, as also emphasized by many other writers in this field (e.g., Bergman & Vargha, 2013; Molenaar, 2004; Richters, 2021). Population-level research and person-level research deal with radically different kinds of research questions – so far, I am in complete agreement with Lamiell (2024). Our disagreement starts with my contention that some population-level research still belongs within psychological science.

### 2.2. Causality at the population level vs. the person level and mechanism level

Lamiell (2024) raises questions about causality that need to be discussed in more detail. He refers to Harré’s (1981, p. 14) claim that “causal processes occur only in individual beings” because “mechanisms of action, even when we act as members of collectives, must be realized in particular persons”. He therefore concludes that there is no such thing as “population-level effects of various psychological interventions” (Lamiell, 2024, p, 62). One problem with this reasoning is that it rests on *the assumption that statements about causality are necessarily of a mechanistic kind* (i.e., they must involve “mechanisms of action”). In other words, it restricts causality to the mechanism level.

In terms of the three-branch model, we may speak of causality at all three levels. Two examples may illustrate this. The first example is about causality in psychotherapy research, and the other about the causes behind non-suicidal self-injury among adolescents.

#### 2.2.1. Example 1: Causality in psychotherapy research

Examples of causality at the population level are when a certain psychological intervention or a certain risk factor is reported to have the causal power of *increasing the probability of certain outcomes*. This is the kind of causality that is shown in randomized controlled trials (RCTs), which are today typically seen as the golden standard when it comes to provide evidence for the causal efficacy of psychological treatments. At the same time, RCTs have failed to produce knowledge about the *mechanisms* behind these apparently effective treatments. As summarized by Kazdin (2007, p. 1): “after decades of psychotherapy research we cannot provide an evidence-based explanation for how or why even our most well-studied interventions produce change”. In other words: this research typically produces evidence *that* a treatment is causally effective (which is also a question about causality), without providing any understanding of *why* it is effective.

Moreover, RCTs as such have little to say about the question *for whom* an evidence-based treatment is effective, and why it is effective for *some* individuals and *not* for others. These are questions at the person-level, which RCTs do not provide any answer to. In other words: RCTs provide information only about population-level causality (*that* something is causally effective), but neither about person-level causality (e.g., *what* is effective in each specific treatment) nor about mechanism-level causality (the underlying mechanisms of change). If RCTs were *combined* with idiographic analyses of the treatment processes of individual patients, however, it might help to illuminate causal processes at the person-level. As pointed out by Hofmann et al. (2024)

a process-based approach, with intensive and frequent assessment linked to a modern time series and network analysis can augment randomized clinical trials, fostering the research program’s sensitivity to the individual while nomothetic questions are examined, without violating logical and statistical assumptions. (Hofmann, 2024, p. 65-66)

#### 2.2.2. Example 2: The causes of non-suicidal self-injury among adolescents

To take another example, the three different kinds of questions about causality can also be illustrated by research on non-suicidal self-injury (NSSI). NSSI is a phenomenon that has apparently become increasingly more common among adolescents during the last decades. Consider the following four questions about what causes NSSI:

1. Why has NSSI increased among adolescents during the last decades? This is a question about causality at the *population* level, which can be approached by means of either sociological or psychological methods.
2. Why do some adolescents engage in NSSI, whereas others don’t? This is a question in *population-level psychology* that has commonly been approached by research on *risk factors* that predict the development of NSSI. Research indicates, among other things, that depressive symptoms (e.g., Lundh et al., 2011) and a negative body image (Black et al., 2019) are such risk factors.
3. What makes an individual person engage in specific instances of NSSI? This is a question at the *person* level, that asks for personal feelings, intentions, and beliefs that motivate an individual to self-injure at specific moments in time. Research using ecological momentary assessment to assess in vivo moment-to-moment feelings indicates that individuals often engage in NSSI as a form of emotion regulation, and that the emotion being regulated often is anger at oneself (e.g., Armey et al., 2011; Nock et al., 2009; Kranzler et al., 2018).
4. Self-injury is painful; why doesn’t the pain stop these individuals from injuring themselves? This can be interpreted as a question at the *mechanism* level. Laboratory studies provide a possible explanation by showing evidence of a degree of pain analgesia in both adults and adolescents who engage in NSSI, in the form of a higher pain threshold and higher pain endurance (e.g., Glenn et al., 2014).^1^

The main point here is that *different questions about causality are studied at the three different levels of psychological research*, and that all these questions are relevant and important. It should be noted that neither at the population-level nor at the person-level is there a search for “mechanisms of action” (which, according to Lamiell, is what causality is about). At the population level the search is for risk factors that can be established by means of longitudinal designs. At the person-level the search is for feelings, intentions, and beliefs that can be explored, for example, by means of experiential sampling or ecological momentary assessment. (For a more detailed discussion of causality at different levels of analysis, se section 5.)

#### 2.2.3. Population-level research in demography, psychology, and other sciences

Lamiell (2024) argues that psychological research at the population level, whether correlational or experimental, is inherently *demographic* and *not* psychological in nature. Against this claim, I will argue that:

- population-level research takes place in various sciences apart from demography, and what makes the difference is the *research questions* that are involved; and
- to the extent that the research questions are of a *psychological* kind, research at the population level belongs to psychology rather than demography.

Demography is commonly defined as the statistical study of populations in terms of their size, composition (e.g., age, ethnicity) and how they change due to fertility, mortality, and migration (e.g., McFalls, 2007). These are core research questions within demography. Sociology is probably the science that is most closely associated with demography. In social demography, a major focus is on the relation between social factors (e.g., the levels and distribution of income, levels of education, or the position of women) and demographic features such as fertility, mortality, and migration.

Population-level research involving other research questions have a central role in a range of other sciences. One illustrative example is genetics. Consider, for example, the following description of population genetics:

Population geneticists develop abstract mathematical models of gene frequency dynamics, extract predictions about the likely patterns of genetic variation in actual populations, and test the predictions against empirical data… Population genetics is intimately bound up with the study of evolution and natural selection, and is often regarded as the theoretical cornerstone of evolutionary biology. This is because “evolution” has traditionally been defined as any change in a population’s genetic composition (Okasha, 2024)

No one would probably claim that population genetics is a branch of demography and *not* of genetics. This illustrates how population-level research does go on in other sciences than demography. So why should it not have a place also in psychological science, especially *if the research questions are about the association between various psychological phenomena*?

Consider the following example: In a longitudinal study, Foster et al. (2024) found that 13-15 years old adolescents who were dissatisfied with their bodily appearance showed a significantly higher risk of developing disordered eating over the following ten-year period. This is a finding at the population level that is relevant to the understanding of possible causes behind the development of disordered eating. Body dissatisfaction and disordered eating clearly are psychological rather than demographic phenomena, and the study of the longitudinal associations between these represents an example of psychological research at the population-level.

The problem with population level research in psychology starts when it is used to draw conclusions at the level of the individual. Lamiell (2024, p. 62) makes an important point when he argues that it is still too often believed that population-level research “offers *an alternative means to the same overall knowledge objective*” as person-level research. It is important to realize that research at these two different levels provides very different kinds of knowledge, but that *both kinds of knowledge are important to the development of psychological science*.

This is probably the main difference between the three-branch model and Lamiell’s position. As argued by Lamiell, the ultimate knowledge objective of psychology as a discipline is “the knowledge of individual-level realities” (Lamiell, 2024, p. 62). According to the three-branch model, person-level knowledge objective may represent the *core* of psychological science, but the knowledge of population-level associations between psychological phenomena *also* represents an important form of psychological knowledge.

## 3. The Three-Branch Model is not as Clear-Cut as it Might Seem

Another critique of the three-branch model is raised by Nilsson (2024), who argues that the division of psychological science into three branches or subdisciplines is not as clear-cut as it might seem. This is an interesting point. In fact, the three-branch model was never intended to describe three neatly divided fields of research. There clearly are various overlaps and interactions between measurements, analyses, and conceptualizations across these three fields, although this was insufficiently expressed in my previous paper.

The division between the three branches can be seen on analogy with a similar subdivision in the science of genetics. The suggestion of the three-branch model in the previous paper (Lundh, 2023) was inspired by a similar subdivision in genetics. As described by Pierce (2020), the science of genetics consists of three major subdisciplines: classical genetics (transmission genetics), population genetics, and molecular genetics. In classical genetics, the focus is on the *individual organism* and how the individual inherits its genetic makeup and passes its genes on to the next generation. In population genetics the focus is on the genetic composition of *populations* of individuals, and how that composition differs geographically and changes with the passage of time. Finally, in molecular genetics the focus is on *molecular processes* within the individual, such as cellular processes of replication, transcription, and translation (by which genetic information is transferred from one molecule to another) and gene regulation (the processes that control the expression of genetic information). At the same time, Pierce (2020) states very clearly that:

Division of the study of genetics into these three subdisciplines is convenient and traditional, but we should recognize not only that they overlap but also that each one can be further divided into a number of more specialized fields, such as chromosomal genetics, biochemical genetics, and quantitative genetics. (Pierce, 2020, p. 6)

If we keep to genetics as a model for the three branches of psychological science, to explore where this may lead us, we should expect the same to apply for psychological science: that is, the three suggested branches should be expected (1) to show various overlaps, and (2) to be possible to subdivide into more specialized fields.

One illustration of this kind of overlap, which at the same time gives an example of a subdivision into more specialized fields, is the differentiation that can be made between two varieties of person-oriented research: *personal profile research* (using methods such as cluster analysis and latent profile analysis; see Bergman & Magnusson, 1997 for an overview) and *person-specific research* (e.g., involving time-series analyses of individual development over time; e.g., Molenaar & Campbell, 2009). Of these, personal profile research involves an overlap between person-level and population-level research; as described in my previous article (Lundh, 2023, p. 78) it can be seen as a person-oriented form of population-level research, because what is studied here are *subgroups of populations*, defined in terms of personal profiles on a set of variables.

As pointed out by Nilsson (2024), this type of research is typically based on the use of population-based statistics (e.g., factor analysis) to define the units of the patterns defining these profiles:

the units of the patterns—that which they vary across—are typically scores on variables that were not designed to capture individuality. In fact, the appropriateness of the variables is often justified in terms of evidence procured through factor analysis and other kinds of population-based statistics … which capture systematic differences *between* individuals rather than properties *within* individuals (Nilsson, 2024, p. 57).

As Nilsson recognizes, this does not mean that these methods are illegitimate. On the contrary, he admits that “they can to some extent be useful for procuring knowledge about individuals even though they involve population-based statistics” (Nilsson, 2024, p. 57).

At the same time, Nilsson (2024, p. 57) makes an important point when he adds that “if we want a more complete understanding of psychological phenomena as they are experienced and enacted by individuals”, we have to go beyond population-based statistics and search for conceptualizations and measurements that are more “sensitive to the psychological meaning and structure of properties of individuals and their environments” (p. 57). Here he introduces the concept of *person-sensitivity* as an alternative to the conceptualization of psychological research in terms of the three-branch model.

Person-sensitivity is an interesting concept, which deserves to be analyzed in more detail. Here I will argue that it represents a *complement* rather than an alternative to the three-branch model. The remaining part of this paper is divided into two main sections, section 4 and 5. In section 4, I will argue that the person/population contrast involves a variety of methodological approaches, both at the population level and at the person-level, which involve various *degrees of person-sensitivity* and correspondingly more circumscribed forms of *generalizability*. Degree of person-sensitivity can be seen as inversely related to the extent of generalizability. The highest degree of person-sensitivity is found in research with the most circumscribed form of generalizability – within-person generalizability.

Section 5 is devoted to the person/mechanism contrast. In contrast to the person/population contrast, which is about methodological approaches, the person/mechanism contrast is about theoretical focus, stretching from simple sub-personal mechanisms to the complex interactions that characterize whole-person functioning.

### 3.1. Methodological and theoretical focus on persons

To summarize, this means that the three-branch model can be depicted in a four-field table of different forms of research (see Table 1), with the rows representing type of *methodological* focus (individual person or population) and the columns representing type of *theoretical* focus (sub-personal mechanisms or whole-individual functioning).

**Table 1:** Methodological focus and theoretical focus in the three-branch model, as exemplified by different forms of research.

		*Theoretical focus*	
		*Sub-personal mechanisms*	*Whole-person functioning*
***Methodological focus***	***Individual person***	Case studies in neuroscience Experimental behavior analysis (operant and respondent paradigms)	Person-specific research Narrative analysis
	***Population of individuals***	Twin studies and adoption studies to establish genetic and environmental influences on personality	Personal profile analysis Longitudinal studies of the interaction between parents and children

In Table 1, person-level research in its most pure form is found in the upper right quadrant, which includes research focused on *whole-person* functioning in *individual persons*. Person-oriented research can, however, also be found in the lower right quadrant (which combines a theoretical focus on whole-person functioning with a methodological focus on populations) and in the higher left quadrant (which combines a methodological focus on individual persons with a theoretical focus on sub-personal mechanisms). In addition, we may find finer gradations (for example in degree of person-sensitivity) within each of the four quadrants in the table.

## 4. The Person Level as Contrasted with the Population Level

Nilsson’s (2024) notion that research can differ in degree of person-sensitivity is clearly compatible with the reasoning in my previous paper (Lundh, 2023). As stated in that paper, there *is* a clear difference between population-level research, which *starts at the population level* and tries to generalize either about this population or about subgroups from this population, and person-level research which *starts at the level of individual persons* and tries to generalize either within individual persons or across a series of individuals. Yet it is possible to make finer gradations within each of these two levels, where increasing person-sensitivity corresponds to more circumscribed forms of generalizability. First, I will discuss this at the population-level and then at the person-level.

### 4.1. Person-sensitivity in population-level research

To extend and refine the arguments from the previous paper, it seems possible to differentiate between at least three degrees of increasing person-sensitivity, corresponding to more circumscribed forms of generalization, in population-level research:

1. The lowest degree of person-sensitivity is found in pure *correlational* variable-oriented research. Here the focus is not on populations of individuals but on “relations between values on variables in the population” (Lundh 2023, p. 78), as studied for example, by correlational methods, multiple regression, or structural equational models. This focus on relations of variables to other variables means that the individual person tends to disappear from the picture, thus indicating a very low person-sensitivity. Still, this is an adequate approach in some forms of research, as for example in psychometric research aimed at understanding the factor structure of psychometric instruments and their construct validity (as seen in their structure of correlations with other measures).
2. At least some degree of person-sensitivity is seen in survey research where the research questions are about the *prevalence* of various kinds of psychological phenomena, such as mental disorders and changes in their prevalence (epidemiological research). Another example is randomized controlled studies (RCTs) of psychological treatments that report the percentage of patients who show remission or clinically significant improvement. What gives these kinds of research a certain degree of person-sensitivity is that they report the percentage *of individuals* who share a certain characteristic, or who respond to a certain treatment. That is, the population is dichotomized into subgroups.
3. More of person-sensitivity is found in studies that identify and compare three or more subgroups with different *personal profiles* on some set of variables in a population. Here the aim is to identify subgroups (classes, categories, types) of individuals with different ways of functioning in some respect, where the intention is to generalize across individuals within each subgroup. There are many different forms of personal profile analysis, which differ not only in how the profiles are identified, but also in how they are classified into subgroups, and in how these subgroups are further analyzed in relation to other data. In the following subsections, three different varieties of personal profile analysis are briefly described: latent profile analysis, cluster analysis, and Q-methodology.

#### 4.1.1. Latent profile analysis (LPA)

Among methods for personal profile analysis, a comparatively low degree of person-sensitivity is often found in studies that make use of model-based methods such as latent profile analysis and latent class analysis. There are several reasons for such a conclusion. First, the individuals are classified into subgroups based upon membership probabilities estimated from models derived from population-based statistics (e.g., Spurk et al., 2020). Second, such studies often lead to the identification of merely 3-4 subgroups. Third, these subgroups are often defined in terms of the *degree* to which the participants possess a set of traits (e.g., “low, “medium”, “high”, and sometimes “very high”); in that case, the results can be questioned as to how much they add to a mere correlational approach.

On the other hand, these methods may have a larger potential, as illustrated by Spurk et al.’s (2020) discussion of a study of vocational behaviour, where they used measures of working compulsively, working excessively, and work engagement, and conducted LPA based on these three indicators:

Both the three- and the four-profiles solutions showed profiles that differed only in the overall level of the three indicators… and did not offer substantive interpretations of much theoretical interest (e.g., no qualitatively different models emerged). Therefore, we continued the examination of additional solutions, and finally decided the eight-profiles solution to be the most appropriate. (Spurk et al., 2020, p. 13-14)

In this study, the eight groups with different profiles were compared on outcome variables such as burnout, and apparently produced results with a higher degree of person-sensitivity than found in many other LPA studies.

#### 4.1.2. Cluster analysis

Among methods for personal profile analysis, comparatively higher degrees of person-sensitivity may be seen in research that uses cluster analysis to identify subgroups with different personal profiles on a set of variables. There are several reasons for such a conclusion. First, this kind of research strives for a more differentiated set of subgroups; according to Bergman (1998) the number of clusters should not be expected to be less than five, although not more than 15.

Second, it is not assumed that all individuals can be classified (Bergman, 1988); the analysis therefore starts by separating a set of outliers into a “residue” of individuals to be analyzed separately. Although some of these individuals may be outliers because of measurement errors, others may represent “true, unique patterns" or “almost unique patterns of values, and these individuals should not be combined with individuals represented by the common patterns” (Bergman, 1988, p. 427).

Third, at least some varieties of cluster analysis start at the person-level. One example is Wards’s hierarchical clustering method, which starts by considering each individual case as a separate cluster; at each subsequent step in the analysis, the two clusters are then merged that result in the smallest increase in the overall error sum of squares (ESS). All this makes this methodology into something akin to a “bridge” between person-level and population-level research.

Advanced methods for cluster analysis have previously been available primarily in less user-friendly statistical packages. During the last decade, however, this situation has changed with the development of ROPstat (Vargha et al., 2015) and the freely available ROP-R (Vargha & Grezsa, 2024).

#### 4.1.3. Typologies, taxometrics, and “carving nature at its joints”

A unique value of methods for personal profile analysis is their potential ability to identify types, discrete classes, or categories of patterns that might “carve nature at its joints” (Gangestad & Snyder, 1985). Too many typologies and classification systems in psychology and psychiatry have been developed on shaky grounds, and several writers have expressed the hope that taxometric methods may improve this situation. For example, writing about the classification problem in psychopathology, Meehl (1995) argued that, “Further revision of diagnostic systems should be based on taxometric analysis rather than on committee decisions based on clinical impressions and nontaxometric research.” Similar arguments for the identification of types within the field of development psychopathology were put forward by Bergman and Magnusson (1997) when they argued that,

Although there is, theoretically, an infinite variety of differences with regard to process characteristics and observed states at a detailed level, there will often be a small number of more frequently observed patterns (common types), if viewed at a more global level. The assumption is made both intraindividually (viewed over time for the same person) and interindividually (for different individuals at the same time or over time). (Bergman and Magnusson, 1997, p. 293)

They carry this argument further by arguing for a search not only for *types* (i.e., patterns that occur significantly more often than expected by chance) but also for *antitypes* (e.g., patterns that occur significantly *less often* than chance; von Eye, 1990), or so-called “white spots” (Bergman & Magnusson, 1997, p. 309), that is, developmental paths that rarely occur. As formulated by Bergman et al. (2009, p. 989), “What does not happen fences what does happen and both aspects should be taken into account when explaining development.”

#### 4.1.4. Q-methodology

Common to the methods of personal profile analysis mentioned so far – LPA and cluster analysis – is that the personal profiles are analyzed as *patterns of values on variables*. As pointed out by Nilsson (2024), there are also other varieties of person-profile analysis that do not make use of variables or population-based statistics. One example is Q-methodology, which was developed by Stephenson (1953) to study people’s subjective viewpoints (ways of thinking).

In this methodology, participants are presented with a set of materials (e.g., verbal statements) which they are asked to judge (e.g., the degree to which they agree with these) by sorting the materials into a set of piles numbered, for example, from -3 (Agree least) to +3 (Agree most); the participants are typically asked to place a fixed number of materials in each category (McKeown & Thomas, 2013). The data are then subjected to a Q factor analysis which groups together *individuals with similar response patterns*. In this way the many individual viewpoints of the participants are reduced to a few factors which are claimed to represent shared ways of thinking. Churruca et al. (2021) report evidence of increased use of Q-methodology in healthcare research.

#### 4.1.5. Summary

- All kinds of population-level research *start* from an analysis of populations (or groups), although it may also analyze these into subpopulations (or subgroups).
- Different methodological approaches in population-level research differ in degree of *person-sensitivity*. The highest degree of person-sensitivity is found in various forms of *personal profile analysis*.
- There are many different varieties of personal profile analysis, of which some (or some applications of these) may be more person-sensitive than others.
- A possibly unique value of personal profile analysis is its potential ability to identify discrete classes, types and antitypes, or categories of patterns that might “carve nature at its joints”.

### 4.2. Person-sensitivity in person-level research

One important implication of Nilsson’s (2024) reasoning is that even person-level research can differ in degree of person-sensitivity. It is not entirely easy, however, to classify the various kinds of research methodologies that are used at the level of the individual into mutually exclusive categories, nor to rank them in terms of their degree of person-sensitivity. Instead, the following section contains a provisional categorization under three subheadings – case studies, person-specific research, and qualitative research. In fact, each of these “categories” contains a wide variety of research types; in addition, these categories may also overlap in various ways. The main point to be illustrated by the following examples is that there is a considerable variety of research methods at the person-level, but that they all *start from a study of individuals* before eventual generalizations.

#### 4.2.1. Case studies, and theoretical generalization from a series of case studies

Case studies have traditionally played an important role in neuroscience. To take a prominent example from the history of neuropsychology: In 1861, the French surgeon and anthropologist Broca described the case of a man who had suffered a stroke and lost the ability to speak fluently, although he could still understand spoken language. As summarized by Kandel (2006):

At the postmortem examination Broca discovered a damaged area, or lesion, in a region of the frontal lobe now called Broca’s area… He went on to study, after their deaths, the brains of eight other patients who had been unable to speak. Each had a similar lesion in the frontal lobe of the left cerebral hemisphere. Broca’s findings provided the first empirical evidence that a well-defined mental capacity could be assigned to a specific region of the cortex. (Kandel, 2006, p. 122)

Here we have an example of how *a series of case studies* are used to draw conclusions about *people in general*. This is different from the examples of population-level research that were described above, which start from an analysis of populations rather than individuals and makes use of statistical tools for generalization.

#### 4.2.2. Person-specific research and idiographic networks

Person-specific research (e.g., Molenaar & Campbell, 2009) uses methods such as experiential sampling and ecological momentary assessment to collect *intensive longitudinal data*, together with methods such as time series analysis and network analysis to analyze these data. Here what is searched for primarily are patterns *within individuals*, although there is also an ambition to generalize into patterns *across individuals*. New methods have also been developed for the purpose of simultaneously modeling idiographic (i.e., person-specific) processes and a nomothetic (i.e., general) structure from intensive longitudinal assessments; one such method is GIMME (group iterative multiple model estimation; Gates & Molenaar, 2012). Although GIMME was first developed in the context of brain research on connectivity mapping, it has also been applied to research on personality and psychopathology (e.g., Wright et al., 2019).

Network analysis has recently gained much attention in research on psychopathology and psychotherapy (e.g., Borsboom, 2017; Fisher et al., 2017). In contrast to the latent-disease model of psychiatric nosology, this approach aims to analyze psychopathology at the level of the individual as networks of symptoms that interact in a way that stabilizes the problem. The relations between symptoms are graphically represented in networks where symptoms are the “nodes” and causal inter-symptom relations are the “edges”. As Hayes et al. (2019) exemplifies,

Such dynamic networks can convey idiographic information about how certain processes unfold over time for each individual… The temporal network structure might reveal that certain nodes (e.g., fatigue) prospectively predict other nodes (e.g., rumination) at a later time point. (Hayes et al., 2019, p. 46).

Network analysis is also the method focused on by Hofmann et al. (2020; 2024) in their description of process-based psychotherapy:

treatment is a dynamic process involving numerous variables that may form bi-directional and complex relationships that differ between individuals. Such relationships can best be studied using an individual dynamic network approach connected to nomothetic generalization methods that are based on a firm idiographic foundation. (Hofmann et al., 2020, p. 1)

Importantly, these methodological approaches all *start* by attempting to find generalizable patterns *within* the individual, that can then eventually be generalized *across* individuals.

#### 4.2.3. Studies with qualitative methods

There are many different forms of qualitative methods, of which some are person-oriented and others are not. One of the most popular qualitative methods in present-day psychological research is thematic analysis (Braun & Clarke, 2006); this, however, is not a person-oriented method, as it does not focus on individual persons but on *themes across persons*.

Other qualitative methods, such as narrative analysis, have a clear focus on individual persons. Sarbin (1986), who coined the term “narrative psychology”, argued that stories are useful to understand human conduct. According to McAdams’ (2001) life story model of identity, “people living in modern societies provide their lives with unity and purpose by constructing internalized and evolving narratives of the self” (p. 100). As in other methodological approaches at the person-level, the study of life stories starts with the individual person, and then eventually generalizes across individuals. As McAdams exemplifies, life stories can be compared and contrasted with respect to the salience of various themes such as agency versus communion, and also with respect to formal characteristics such their structural complexity, coherence, and intelligibility.

Nilsson (2024) makes several interesting comments that add to this picture. One is that there are a variety of *mixed* methods, which combine qualitative and quantitative methods. Another is Nilsson’s suggestion that the highest level of potential person-sensitivity might be found in research that attempts to *tailor* the interpretive framework to the subject’s worldviews, experiences, and actions through in-depth qualitative interviews. Two additional points made by Nilsson (2024) are that it might be useful to differentiate between *various aspects of person-sensitivity*, such as (1) person-sensitive conceptualizations, (2) person-sensitive measurements, and (3) person-sensitive analyses; and that there may be room for further development particularly with respect to the role of person-sensitivity in conceptualization and measurement. These are topics that should be pursued in further analyses.

#### 4.2.4. Summary

- All kinds of person-level research *start* from an analysis of individuals, and then eventually generalizes from individuals to larger groups.
- There are many different varieties of personal level research, of which some (or at least some applications of these) may be more *person-sensitive* than others.
- Person-level research includes studies *both* of sub-personal mechanisms (e.g., case studies in neuro-science) *and* of whole-person functioning (e.g., experiences of identity, as studied in narrative analyses of life stories).

## 5. The Person Level as Contrasted with the Mechanism Level

The person/mechanism distinction differs from the person/population distinction by contrasting different *theoretical,* rather than *methodological,* levels of research. At the mechanism level, the focus is on *sub-individual* mechanisms. At the person-level the focus is on *whole individuals and their causal capacities*. At both levels, it is possible to differentiate between various *degrees of causal complexity*. At the mechanism level we find not only mechanisms of varying complexity, but also more or less complex interlockings between different mechanisms. At the person level we find individuals with more or less complex causal capacities. In the following, it is argued that the connection between the two levels takes the following form:

Increasingly more complex *mechanisms*, and increasingly more complex *interlockings* between mechanisms, allow for the emergence of *individuals with increasingly more complex causal capacities*.

Of course, there is nothing that says that increasing complexity will always be most adaptive during evolution. Still, it can hardly be denied that evolution has led to the development of species of individuals with increasingly more complex capacities.

What we call *persons* are individuals with possibly the most complex combination of causal capacities that have been seen so far. Peter Ossorio (2006) and Christian Smith (2010) have suggested possible definitions of what characterizes persons, and their suggestions are briefly discussed below. Other researchers who have written about relevant topics in this context are Mark Bickhard (2016) and Terence Deacon (2012), although with a primary focus on less complex individuals. The purpose of the following discussion is to point to some important topics in this area that deserve further discussion and more detailed analyses.

The present section is divided into two subsections. First, in section 5.1, various kinds of research on sub-personal mechanisms are described and compared in terms of causal complexity. Then, in section 5.2, the corresponding questions are discussed with regard to research into whole-person functioning.

### 5.1. Causal complexity in mechanism-level research

Causal mechanisms relevant to psychological functioning can be found in many different forms and at many different levels. Here three categories of mechanisms are briefly discussed: neural mechanisms, genetic mechanisms, and psychological mechanisms.

#### 5.1.1. Neural mechanisms

At an elementary level of the neural system, we find specialized fields such as *molecular* neuroscience and *cellular* neuroscience. The causal processes here represent basic mechanisms common to animals of many species. An illustrative example is Kandel’s (2006) research on the molecular mechanisms that underlie basic forms of memory and learning, for which he was awarded the Nobel Prize in 2000. This research was carried out on *Aplysia*, a snail with only around 20,000 nerve cells (as compared with the about 100 billion neurons found in the human brain), which made it into a suitable animal for this kind of laboratory research. Importantly, Kandel’s research showed that short-term memory involves a transient strengthening of pre-existing synaptic connections between neurons, whereas long-term memory involves the growth of new synaptic connections.

The relevance of these findings to human experiences and skills is seen, for example, in research that show brain changes as the result of learning. For example, brain imaging of the somatosensory cortex in violinists and cellists show that the area of the cortex devoted to the fingers of the left hand (which string players use to modulate the sounds of the strings) are much more extensive than those in non-musicians. There are no corresponding differences in the areas devoted to the fingers of the right hand, which move the bow and are not involved in such highly differentiated movements. As Kandel (2016) points out, “the architecture of each person’s brain is unique. Even identical twins with identical genes have different brains because of their different life experiences” (p. 217).

At increasingly “holistic” levels of analysis, the neural system involves successively more complex forms of mechanisms, and more complex interlocking between different meachanisms. Bear et al. (2016), in their textbook on neuroscience, differentiate between the following levels above the level of molecular and cellular neuroscience:

- *systems* neuroscience, where the focus is on constellations of neurons that form complex circuits underlying various functions such as, for example, vision or voluntary movement.
- *behavioral* neuroscience, which is the study of how neural systems work together to enable integrated behaviors and actions.
- *cognitive* neuroscience, where the focus is on understanding the neural mechanisms responsible for higher levels of mental activity such as self-awareness, imagination, and executive functioning.

Although this division of neuroscience into subspecialties takes us from “atomistic” to increasingly more “holistic” levels of analysis, all these kinds of research remain at a sub-personal mechanism level. This is also true of the study of genetic mechanisms.

#### 5.1.2. Genetic mechanisms

As summarized by Dawkins (1976, 1989, 2016), genes do primarily two things: (1) they replicate themselves, and (2) they act on their environment via the protein synthesis. This can be seen as representing two very different levels of sub-personal mechanisms.

- In their capacity as *replicators*, the genes pass on their structure largely intact in successive replications, with change occurring only slowly due to random mutations. The genes thereby bring continuity to life. As Dawkins (2016) describes it, genes are potentially “immortal”, as distinct from individual organisms which are deadly creatures with a limited life span. This represents processes very far away from the person-level.
- Genes act on their environment by constructing proteins, which in turn have effects on other processes in a long causal chain which end up in specific phenotypes. In contrast to the replication process, which is “inflexible apart from the rare possibility of mutation” Dawkins (1989, p. 21), this interaction process is “exceedingly flexible”.

Arguing against genetic determinism, Dawkins emphasizes that “the variance we seek to explain will have many causes, which interact in complex ways… Environmental events, both internal and external, may modify the effects of genes” (p. 19). Importantly, the expression of genes is critically dependent not only on the larger environment but also on each other:

Sometimes a gene has one effect in the presence of a particular other gene, and a completely different effect in the presence of another set of companion genes. The whole set of genes in a body constitutes a kind of genetic climate or background, modifying and influencing the effects of any particular gene. (Dawkins, 2016, p. 47)

In view of this “intricate interdependence of genes”, where “it is almost impossible to disentangle the contribution of one gene from another” (Dawkins, 2016, p. 29), Dawkins poses the question why we need to speak of *individual genes* at all. His answer is that we need to assume the existence of individual genes because of our sexual reproduction with its crossing-over process during meiosis, which “has the effect of mixing and shuffling genes” (p. 30) so that each new individual who is born is characterized by a uniquely new combination of genes. Importantly, this means that the same gene may have completely different effects in different persons depending on the specific combination of genes that characterizes the individual, and its interaction with the environment. In other words, these processes involve much more complex mechanisms than the genetic replication process (see also Lundh, 2021).

#### 5.1.3. Psychological mechanisms

Neural and genetic mechanisms can be seen as residing within the body of the individual. Psychological mechanisms, on the other hand, also involve mechanisms in the interaction between individuals and their environment. A simple form of causal mechanism of this kind is the stimulus-response mechanism, where a stimulus automatically elicits a response. A slightly higher degree of complexity is found in classical respondent conditioning, where a new stimulus acquires the ability to elicit a certain response by being paired with it.

An even more complex mechanism is seen in what Skinner (1938, 1969) refers to as operant conditioning, where a stimulus acquires the ability to *increase the probability of* a certain behaviour because of the consequences that follow. Whereas the causal mechanism in respondent behaviour is depicted by the simple formula S → R (i.e., one type of stimuli eliciting a type of response), operant behavior is captured by the more complex formula S^D^ → R → ^SR^, where S^D^ refers to a discriminative stimulus that “sets the occasion” for an operant behaviour (R) by increasing its probability as a function of the consequences that follow (the reinforcing stimulus, S^R^).

Operant conditioning represents a more complex mechanism than respondent conditioning, in the sense that it (a) involves an organism’s active behaviour in contrast to the merely reflex-like responses that characterize respondent conditioning, and (b) is formed by the consequences that have followed it in the individual’s past, rather than being automatically elicited by a stimulus. Yet, Skinner (1969) assigns no role to experiences, intentions, beliefs, or any other mental processes as part of the causal processes involved. On the contrary, operant conditioning is assumed to be caused by the contingencies of reinforcement in the environment.

Skinner’s radical behaviourism with its respondent and operant paradigms formed the basis of classical behaviour therapy, where each treatment was to be based on a behaviour analysis where the patient’s problems were analysed in respondent and operant terms. An interesting thing about Skinner’s radical behaviourism and its applications, is that it represents a clear example of *idiographic* psychology, although it stays at the *mechanism* level. This thereby gives yet another illustration of how idiographic psychology is possible also at a mechanism level without having to involve phenomena such as experiences, intentions, or beliefs as part of the causal analysis.

#### 5.1.4. Summary

- Studies of sub-personal mechanisms are found in many different areas, such as neuroscience, genetics, and psychology.
- Research in neuroscience shows that the architecture of each person’s brain is unique, and that the anatomy of the brain changes as the result of experience. (e.g., as seen in the growth of new synaptic connections between neurons).
- The sub-personal mechanisms studied in neuroscience and genetics reside within the individual’s body, whereas psychological mechanisms also address aspects of the interaction between individuals and their environment (e.g., as seen in studies of respondent and operant conditioning).

### 5.2. Causal complexity in person-level research

A basic difference between psychological mechanisms as described in section 5.1 and causality at the person level is that the latter involves experiences, intentions, beliefs, and other mental phenomena *as part of the causal process*. According to Magnusson (2001), “a fundamental characteristic and guiding element in an individual’s functional interaction with the environment is consciousness and intentionality, which are linked to values, goals, and emotions” (p. 161). Nilsson (2024) expresses something similar when he speak of persons “as agents with mental states that exhibit intentionality and subjectivity” (p. 58). More detailed definitions are suggested by Peter Ossorio in *The behavior of persons* (Ossorio, 2006) and the sociologist Christian Smith in *What is a person?* (Smith, 2010). From an evolutionary perspective, it is also relevant to broaden the discussion to *individuals* more generally, thus including individuals also from other species than humans.^2^

The purpose of the following section is to sketch very briefly on some themes concerning causal capacities of individuals that might be explored further in more detailed analyses: (1) the ability to act *purposely* based on *perceived* possible interactions with the environment; (2) the ability to act *deliberately* based on a *reflection* about possible interactions before choosing how to act (personal agency); (3) the capacity to relate to others based on *perspective-taking and empathic concern*; and (4) *embodiment* as an integral part of whole-person functioning.

#### 5.2.1. Perception and purposive behaviour

In my previous paper (Lundh, 2023), and inspired by Deacon (2012), I focused on one specific aspect of person-environment interactions: the individual’s ability to relate to *possible* interactions before a real interaction takes place, and to act based on the experience of such possibilities. In the most general sense, this can be exemplified by the perceptually guided interactions with the environment that are described by Gibson (1979) in his ecological approach to perception, where the most important information provided via our senses is about the environment’s *affordances*. Affordances, as defined by Gibson, are what the environment “offers the animal, what it provides or furnishes, either for good or ill” (Gibson, 1979, p 127). As expressed in this quote, this is a kind of individual-environment interaction which humans share with other animals. At the same time, it is an aspect of *whole-individual* functioning.

Perception of affordances is intrinsically related to purposive behaviour, and one basic purpose of living organisms is *self-maintenance* – to avoid dangers, to stay alive, and to reproduce. The concept of *recursive self-maintenance* is central to Mark Bickhard’s (2016) writings on these topics. As he describes it, living systems can maintain their property of being self-maintenant by *shifting* between different processes. Simple forms of recursive self-maintenance are seen even in bacteria and can be seen as mechanisms that have been selected during evolution due to their survival value. At the same time, such mechanisms represent a form of *autonomy* of individuals in relation to their environment, although a very primitive kind of autonomy. As Bickhard (2016) defines it, autonomy is “the ability to make use of environments to maintain persistence of far from equilibrium conditions. Autonomy has to do with how (well) the system manages its interactions for its own persistence” (p. 26). According to Bickhard, this kind of recursive self-maintenance is constitutive of all living systems. As he puts it, living systems are interactive processes that tend to maintain their non-equilibrium conditions, and they do so recursively: “self-maintenance is most fundamentally the system itself maintaining the thermodynamic conditions for its own existence” (p. 27-28).

Partly similar ideas, but on a much more complex level, are seen in Smith’s (2010) characterization of persons as *centers with a purpose*, where the primary purpose is to sustain, nourish and cultivate their selves. In an extension of Smith’s formulations, it is possible to view each living individual (i.e., including animals of many species) as a center with a purpose, where one basic aspect of the individual animal’s center is its *perceptual perspective on the world*, as defined by the position of its body in the environment in combination with the nature of its sensory apparatus.

Typically, we don’t speak about animals as “persons”. Personhood is commonly attributed only to humans and may be assumed to involve much more complex forms of causality than in most non-human animals. Smith (2010) suggests the following definition:

By person I mean a conscious, reflexive, embodied, self-transcending center of subjective experience, durable identity, moral commitment, and social communication who – as the efficient cause of his or her own responsible actions and interactions – exercises complex capacities for agency and intersubjectivity (Smith, 2010, p. 61).

Although any such definition can be criticized either as failing to include some basic characteristics, or as including characteristics that are not sufficiently basic, Smith’s (2010) bold attempt to formulate a general definition of persons should be welcomed as it represents a valuable starting point for further discussion and analysis of these matters.

Smith’ (2010) theory includes the assumption that persons emerge as the result of an interaction between a large set of psychological capacities. Without necessarily agreeing with this assumption, the present paper puts forward the following somewhat similar hypothesis:

Individuals capable of successively more complex forms of perception and purposive behaviour emerge as the result of the development of successively more complex sub-individual mechanisms, and more complex interlockings between sub-individual mechanisms.

In the following, I will briefly discuss some aspects of person-specific characteristics: first self-reflection and agency, then intersubjectivity (perspective-taking and empathic concern), and finally embodiment.

#### 5.2.2. Self-reflection and deliberate action

At a person-specific level of causality, we find the ability to choose action based on a *reflection* about possible interactions with the environment. This may encompass much longer temporal perspectives into the future than interactions with the environment that are merely perceptually guided. As Ossorio puts it, the “appropriate size of the unit for conceptualizing a person is not a behavior but a life history” (p. 384), and a life history where *deliberate action* plays a central role. In Ossorio’s formulation, a person is “an individual whose history is, paradigmatically, a history of Deliberate Action in a Dramaturgical Pattern” (p. 69).

Importantly, deliberate action cannot be reduced to goal-directed or intentional action. When persons engage in deliberate action, as Ossorio (2006) describes it, they know what behavior they are engaging in, in the sense that they can distinguish this behavior from other behaviors and choose to engage in it as distinct from alternative behaviors. In other words, deliberate action differs from other varieties of intentional action by involving *self-reflectiveness*, in the form of a consideration of not only short-term but also long-term ambitions and expectations.

Examples are seen in the development of life projects (e.g., Little, 1983) and in the elaboration of “possible selves” (Markus & Nurius, 1986), defined as an individual’s ideas of what they might become, what they would like to become, and what they are afraid of becoming. Although goals, purposes and expectations are found already at a perceptual level of interaction, the self-reflective level adds ambitions, ideals, and reflection about possible conflicts between competing goals. This is a kind of causality that involves persons as *responsible for their actions* – on the assumption that they could have acted otherwise – and where their choice of action can be causally influenced by various kinds of social incentives and social sanctions.

In present-day psychological research, these kinds of causal capacities are studied under many different labels, such as “controlled information processing” (e.g., Shiffrin & Schneider, 1977), “executive functioning” (e.g., Miyake et al., 2000), and “self-regulation” (e.g., Baumeister et al., 2018). “Controlled information processing” is typically defined as “intentionally initiated sequence of cognitive activities”, and commonly refer to processes that require conscious attention, intention, and effort for the purpose of dealing with novel or complex situations or in the connection of learning of new skills. “Executive functioning” is a more complex construct, described by Miyake et al. (2000) as involving three basic capacities: *updating* (the capacity to monitor one’s short-term memory and quickly add or delete contents), *inhibition* (the capacity to supersede dominant responses), and *shifting* (the cognitive flexibility seen in the capacity to switch between different tasks or ways of thinking). “Self-regulation”, which may be seen as the most over-arching of these constructs, is defined by Baumeister et al. (2018) as “the process by which the self changes its thoughts, feelings, and actions, including impulsive urges and task performance” (p. 141); the concept of “willpower” has a significant role in their model.

Although executive functioning (EF) is sometimes described in terms of cognitive processes that *underlie* individuals’ capacity for self-regulation, it would probably be a mistake to place EF at the mechanism level, in contrast to person-level self-regulation. Morrison and Grammer (2016) speak about the situation in this field of research as suffering from “conceptual clutter” and “measurement mayhem”:

The fuzzy distinction between EF and self-regulation (as well as effortful control)—in combination with existing debates about the measurement of both constructs—has contributed greatly to a proliferation of constructs, resulting in a kind of conceptual clutter whose similarities and differences are not readily apparent. Likewise, for discipline-specific reasons, numerous divergent measures of EF have proliferated, with little consensus on how they relate to each other, which has yielded a similar kind of measurement mayhem. (Morrison & Grammer, 2016, p. 331).

What is needed here are much more detailed theoretical analyses of the various phenomena that are involved, and the development of more precise language, for the purpose of “uncluttering the conceptual landscape” (Morrion & Grammer, 2016, p. 331).

#### 5.2.3. Perspective-taking and empathic concern for others

Just as person-environment interactions in general involve processes both at the perceptual level and at the self-reflective level, this applies also to interpersonal relations. That is, interpersonal relating occurs both at the perceptual level (e.g., perceiving what another person *affords* in terms of relating) and at the self-reflective level, where it can take the form, for example, of perspective-taking and empathic concern for others, skillful cooperation with others, the capacity to influence others by means of skillful communication, but also more sinister forms of social manipulation. This represents an even more complex form of causality than self-reflection and deliberate action in general (as described in section 5.2.2), since these interpersonal interactions are based on the perception of other persons together with a consideration of *their* experiences, intentions and beliefs, and potentially also considerations about how *these* persons apprehend *one’s own* experiences, intentions and beliefs.

As argued by Ossorio (2006; see also Schwartz, 2019) all these kinds of capacities are based on one basic fact: *being a person* means to *have the concept of person, as a basic form of competence*. In other words, intersubjectivity and interpersonal relations involve interactions between two or more persons who are in possession of the concept of person. Ossorio sees this partly on analogy with having the capacity for language. Just as we normally acquire linguistic competence during our psychological development and maturation, we also normally acquire the competence of understanding what it means to be a person. That is, we normally develop an intuitive understanding of other people as having their personal experiences, beliefs, intentions, desires, life projects, etc. It is this that accounts for the basic fact that “people are not inherently mysterious to other people” (Ossorio, 2006, p. 2). As Ossorio formulates it, *mastery of the concept of person* and “the routine spontaneous exercise of that concept is what makes a person a person” (p. 6). To extend the analogy between language and interpersonal competence, it may be argued that just as people are more or less proficient in their use of language, they may also develop various degrees of skill in understanding persons (both others and themselves).^3^

Importantly, interpersonal relations can be studied also at the population level. One example is a study by Khurana et al. (2024), where bidirectional linkages were examined between parenting behaviours (i.e., autonomy support, supportive presence, hostility), and children’s self-regulation and executive functioning (i.e., working memory, inhibitory control) from early childhood to adolescence. This is a kind of research that focuses on the interaction between parents’ and children’s *use of causal capacities*, and not on sub-personal mechanisms. It exemplifies how whole-person functioning may also be studied at the population level (see the lower right quadrant in Figure 1).

#### 5.2.4. Embodiment as an integral part of whole-person functioning

Before leaving this part of the discussion, it needs to be emphasized that whole-person functioning by definition includes all aspects of human psychological functioning and how these interact in various ways. That is, it is insufficient to conceptualize persons only in terms of personal agency, deliberate action, and other conscious processes, without *also* including less intentional and less rational processes into the picture. This is also emphasized by Smith (2010), who attributes an important role to unconscious processes and less rational thinking in whole-person functioning.

Personal being involves certain degrees of internal disconnection, disjuncture and lack of integration between parts… But those disconnections always operate relative what for all normal persons is a more dominant controlling center of coordinated menta and physical activity. (Smith, 2010, p. 62)

Among other things, this also means that the concept of embodiment is an integral part of whole-person functioning (Smith, 2010, p. 63-64). This was clearly expressed already by one of the pioneers in person-oriented research, William Stern (1938), who argued that the concept of *person* is primary to the concepts of body and mind:

Under the personalistic conception the ancient ‘mind-body’ problem receives a new direction, and at the same time loses much of its former significance. The individual is not partly body and partly mind, but a person with the capacity for experience (Stern, 1938, p. 84).

According to Stern, persons have corporeality and mentality, and the life of the person includes both body and mind in the sense that “there is no experience and no capacity for experience that is not bound up with the physical aspect of life and with bodily functions” (Stern, 1938, p. 84).

A central experiential aspect of embodiment is that each person not only *has* a body but also *is* this body (Lundh & Foster, 2024). This puts a wide variety of constraints on each person’s psychological functioning, and at the same time serves as a basic aspect of self-experience that they must relate to in some way. This is a challenge that may be particularly salient during periods when the person undergoes considerable bodily change, such as during puberty or in connection with illness or ageing, which the individual must adapt to somehow. A failure to adapt to these bodily changes may lead to various forms of psychopathology.

On a more fundamental plane, however, *all* everyday psychological functioning relies on bodily functions. For example, memory is sometimes described as “the glue that holds our mental life together” (Kandel, 2018, p.104). Or, in the present perspective: memory mechanisms are fundamental to *whole-person functioning*. There are many different memory mechanisms in the brain, but one of the most basic is the synapses that connect neurons. Alzheimer’s disease results from the loss of synapses. As summarized by Kandel (2018, p. 114), “The brain can regrow synapses in the early stages of disease, but in the later stages, neurons actually die.” Early symptoms of the disease include the forgetting of recent events, but over time the memory problems become more serious and eventually the person loses the ability to perform everyday tasks.

Yet another example of embodiment concerns the interaction between *voluntary* and *involuntary* aspects of whole-person functioning. Personal being involves numerous bodily functions that lie beyond voluntary control, although they interact with other psychological processes in multiple ways. Sleep is a basic bodily function that is not under voluntary control, and insufficient sleep is likely to have detrimental effects on both cognitive functioning and emotional well-being (e.g., Broman et al., 1996). A basic fact about sleep is that it cannot be produced by an effort of the will. Attempts to fall asleep by willful efforts are even likely to backfire by causing frustration and an increased cognitive activation that further delays sleep. Still, it is possible to facilitate sleep *in-directly* by developing a more accepting attitude to the involuntariness of sleep processes, in combination with various practices such as sleep hygiene, specific behavioural routines (“stimulus control”), relaxation exercises, and mindfulness meditation, that are conducive to cognitive deactivation (e.g., Lundh, 2005). In short, psychological processes affect sleep quality, and sleep quality affects psychological functioning, in bidirectional patterns that are typical for whole-person functioning.

#### 5.2.5. Summary

- The hypothesis is that *individuals with successively more complex causal capacities* have emerged as the result of the development of successively more complex *mechanisms*, and successively more complex *interlockings* between mechanisms at a *sub-individual* level.
- Causality at the person level involves experiences, self-reflection, deliberate action, and other mental phenomena *as parts of the causal process*.
- Interpersonal interaction typically requires that the interacting individuals are in possession of *the concept of person as a basic competence*.
- Causality at the person level also involves the effects of non-rational, involuntary, and unconscious processes, that interact with self-reflection and deliberate action.
- Persons are *embodied*. This puts a wide variety of constraints on whole-person functioning.

## 6. Conclusions

One main conclusion from the present analysis is we may speak of several different forms of person-level research, from the “pure” forms of person-oriented research which involve *both* (1) a *methodological* focus on individual persons as distinct from populations of individuals, *and* (2) a *theoretical* focus on whole-person functioning, as distinct from a focus on sub-personal mechanisms. But there is also research that has a *methodological* focus on individual persons, while focusing *theoretically* on sub-personal mechanisms (e.g., case studies in neuroscience). And there is research that has a *theoretical* focus on whole-person functioning while *methodologically* addressing populations rather than individuals (e.g., longitudinal studies of the relation between parents and children).

A second main conclusion is that it may be useful to follow Nilsson’s (2024) lead in speaking of degrees of person-sensitivity of research at both the person-level and population-level. Taken together, all this means that person-level research is a huge field that contains an immense number of different kinds of research, which may differ in the degree to which they are person-oriented, as well as in which aspects they are person-oriented.

Finally, it should be noted that the three-branch model of psychological science as discussed in the present paper is still a rather provisional model, that requires more detailed analysis and elaboration. Although the model has hopefully been at least somewhat clarified in the present paper, largely thanks to the critiques raised by Lamiell (2024) and Nilsson (2024), much more critical discussion and more detailed analyses are required for its further development.

## Footnotes

1 This is an explanation at the *mechanism* level, which involves the assumption that these individuals do not feel pain in a way that stops them from self-injuring. An alternative explanation, however, is in terms of *pain endurance*: maybe these individuals feel pain in the same way as others but are more able to *endure* the pain. There are empirical findings that support such an explanation. First, studies indicate that the enhanced pain endurance is mediated by high self-criticism (e.g., Glenn et al, 2014). And second, a brief intervention targeting self-criticism has been found to normalize the pain endurance temporarily (Hooley & St Germain, 2014). When Hooley and Franklin (2018) summarize these findings, they therefore conclude that “many people who engage in NSSI believe that they deserve pain, and they endure pain longer because of this belief” (p. 440). This is a causal explanation at the *person* level, because it causally attributes these individuals’ behaviour to the *belief* that they deserve pain, in combination with *deliberate action* in the form of a willingness to endure the pain, rather than to a *mechanism* of pain analgesia.

2 In the terminology that is used here, *individual* is a broader concept than *person*, as it includes individuals of all kinds of species. The term “person” is typically applied only to humans, although Ossorio (2006) suggests basic criteria for when it would be appli-cable also to non-human individuals (see also Schwartz, 2014; 2019).

3 In present-day psychological research, these things are often conceptualized in terms of relating to other *minds*, rather than other *persons*. Prominent examples from psychological research are developmental theories about the child’s “theory of mind” (e.g., Baron-Cohen, 2000) and psychoanalytic “mentalization theory” (Fonagy et al., 2002). The concept of *person* is a wider concept than *mind*, as it integrates body and mind (Stern, 1938). Or, in other words: persons are *embodied* (e.g., Ossorio, 2006; Smith, 2010).

## Action editor

Lars R. Bergman served as action editor for this article.

## Open access

This article is distributed under the terms of the Creative Commons Attribution 4.0 International License (http://creativecommons.org/licenses/by/4.0/), which permits unrestricted use, distribution, and reproduction in any medium, provided you give appropriate credit to the original author(s) and the source, provide a link to the Creative Commons license, and indicate if changes were made.
